# Monipar: movement data collection tool to monitor motor symptoms in Parkinson’s disease using smartwatches and smartphones

**DOI:** 10.3389/fneur.2023.1326640

**Published:** 2023-12-07

**Authors:** Luis Sigcha, Carlos Polvorinos-Fernández, Nélson Costa, Susana Costa, Pedro Arezes, Miguel Gago, Chaiwoo Lee, Juan Manuel López, Guillermo de Arcas, Ignacio Pavón

**Affiliations:** ^1^Instrumentation and Applied Acoustics Research Group (I2A2), ETSI Industriales, Universidad Politécnica de Madrid, Madrid, Spain; ^2^ALGORITMI Research Center, School of Engineering, University of Minho, Guimarães, Portugal; ^3^Life and Health Sciences Research Institute (ICVS), School of Medicine, University of Minho, Braga, Portugal; ^4^AgeLab, Massachusetts Institute of Technology, Cambridge, MA, United States; ^5^Escuela Técnica Superior de Ingeniería y Sistemas de Telecomunicación (ETSIT), Universidad Politécnica de Madrid, Madrid, Spain

**Keywords:** mobile health, mHealth, inertial sensors, resting tremor, bradykinesia

## Abstract

**Introduction:**

Parkinson’s disease (PD) is a neurodegenerative disorder commonly characterized by motor impairments. The development of mobile health (m-health) technologies, such as wearable and smart devices, presents an opportunity for the implementation of clinical tools that can support tasks such as early diagnosis and objective quantification of symptoms.

**Objective:**

This study evaluates a framework to monitor motor symptoms of PD patients based on the performance of standardized exercises such as those performed during clinic evaluation. To implement this framework, an m-health tool named Monipar was developed that uses off-the-shelf smart devices.

**Methods:**

An experimental protocol was conducted with the participation of 21 early-stage PD patients and 7 healthy controls who used Monipar installed in off-the-shelf smartwatches and smartphones. Movement data collected using the built-in acceleration sensors were used to extract relevant digital indicators (features). These indicators were then compared with clinical evaluations performed using the MDS-UPDRS scale.

**Results:**

The results showed moderate to strong (significant) correlations between the clinical evaluations (MDS-UPDRS scale) and features extracted from the movement data used to assess resting tremor (i.e., the standard deviation of the time series: *r* = 0.772, *p* < 0.001) and data from the pronation and supination movements (i.e., power in the band of 1–4 Hz: *r* = −0.662, *p* < 0.001).

**Conclusion:**

These results suggest that the proposed framework could be used as a complementary tool for the evaluation of motor symptoms in early-stage PD patients, providing a feasible and cost-effective solution for remote and ambulatory monitoring of specific motor symptoms such as resting tremor or bradykinesia.

## 1 Introduction

Parkinson’s disease (PD) is a neurodegenerative disorder caused by the deterioration of the nerve centers in the brain responsible for movement control (1). PD affects more than 1% of people over 60 years (2). However, due to the aging population, the global prevalence of PD is projected to increase significantly from 6.9 million people in 2015 to approximately 12 million in 2040 (3). PD manifests with a variety of movement-related symptoms, known as motor symptoms, and mental health-related symptoms, known as non-motor symptoms (4, 5).

Currently, there is no cure for PD, and drugs such as levodopa and dopamine agonists remain the most effective treatments to control symptoms (5, 6). The most widely used scale to measure the progression of PD is the Movement Disorder Society-Sponsored Revision of the Unified Parkinson’s Disease Rating Scale (MDS-UPDRS) (7). This scale assesses activities of daily living and psychiatric health using questionnaires and a set of physical tests scored by observation. Although scales such as MDS-UPDRS are commonly used in clinical practice, it is difficult for neuroscientists to assess short-term changes in patients’ symptoms, because PD assessments are usually performed scarcely a year in prescheduled medical appointments. For this reason, clinical visits provide only a brief overview of the patient’s condition, and the subjective nature of clinical tests can lead to biased evaluations (8).

The need for objective evaluation mechanisms in PD has led to the use of technological tools to facilitate management and optimize long-term monitoring (9–12). These tools can improve access to medical care by reducing costs and minimizing physical barriers between patients and medical facilities (13, 14). In specific, mobile health (m-health) technologies, such as wearable and smart devices, present an opportunity to develop clinical tools to support tasks such as early diagnosis, remote monitoring, and objective quantification of symptoms over time (14–17). These technologies can reduce the burden on the patient and provide organized information on the evolution of symptoms (18). Furthermore, data collected by m-health technologies can allow the development of digital biomarkers for the objective quantification of the progression of symptoms and the effects of treatment or therapeutic interventions (19).

The most promising trends in monitoring motor symptoms involve the use of wearable devices (wearables) to capture data from different sensors (i.e., inertial, bioelectrical) (14, 16, 17, 20). Furthermore, recent trends in PD monitoring include the use of research-grade wrist devices (21–23), and smart technologies such as smartphones (SP) (15), and commodity smartwatches (SW) (24, 25) to present promising cost-effective solutions for data collection and monitoring.

Several studies have introduced platforms to detect and monitor motor symptoms. For example, PD_manager (26), utilizes a smartphone (SP) in combination with watch-like sensors and insoles to collect data. Similarly, mPower (27) employs a smartphone for extensive remote data collection, focusing on a range of motor, memory, and voice activities. Another initiative, CloudUPDRS (28), uses a smartphone application to evaluate motor function through gait analysis and physical exercises. Furthermore, i-PROGNOSIS (29) implements several tests for PD early detection through the daily interaction of the patient with his or her SP, collecting data on mood, motor competence, and speech.

Despite the potential of wearables in the monitoring of PD, the implementation of these technologies in clinical practice faces several challenges, such as the lack of standardization on technological platforms, the type of data acquired, and how they are managed (30). Furthermore, there is no clear consensus on the number of sensors or the best place to place them on the body, as it is convenient to use the minimum number of units to facilitate usability, portability, and comfort, without affecting the quality of the information collected (31, 32). Additionally, few studies have reported evidence on the capability of off-the-shelf SW to collect accelerometer data remotely to assess specific motor symptoms.

In this context, this study evaluates the potential of a monitoring framework to derive useful data to monitor motor symptoms. The proposed framework uses an *ad-hoc* m-health tool named Monipar to acquire movement data in combination with a monitoring protocol based on the performance of a set of standardized exercises. In specific, Monipar uses off-the-shelf smart devices (i.e., SW and SP) to collect accelerometer data during the execution of guided movement tasks. Furthermore, to identify the potential of the framework, a database was collected, curated, and used to extract relevant indicators to assess some motor symptoms. Finally, these indicators were compared with clinical evaluation.

## 2 Materials and methods

### 2.1 Overview

**Figure 1:**
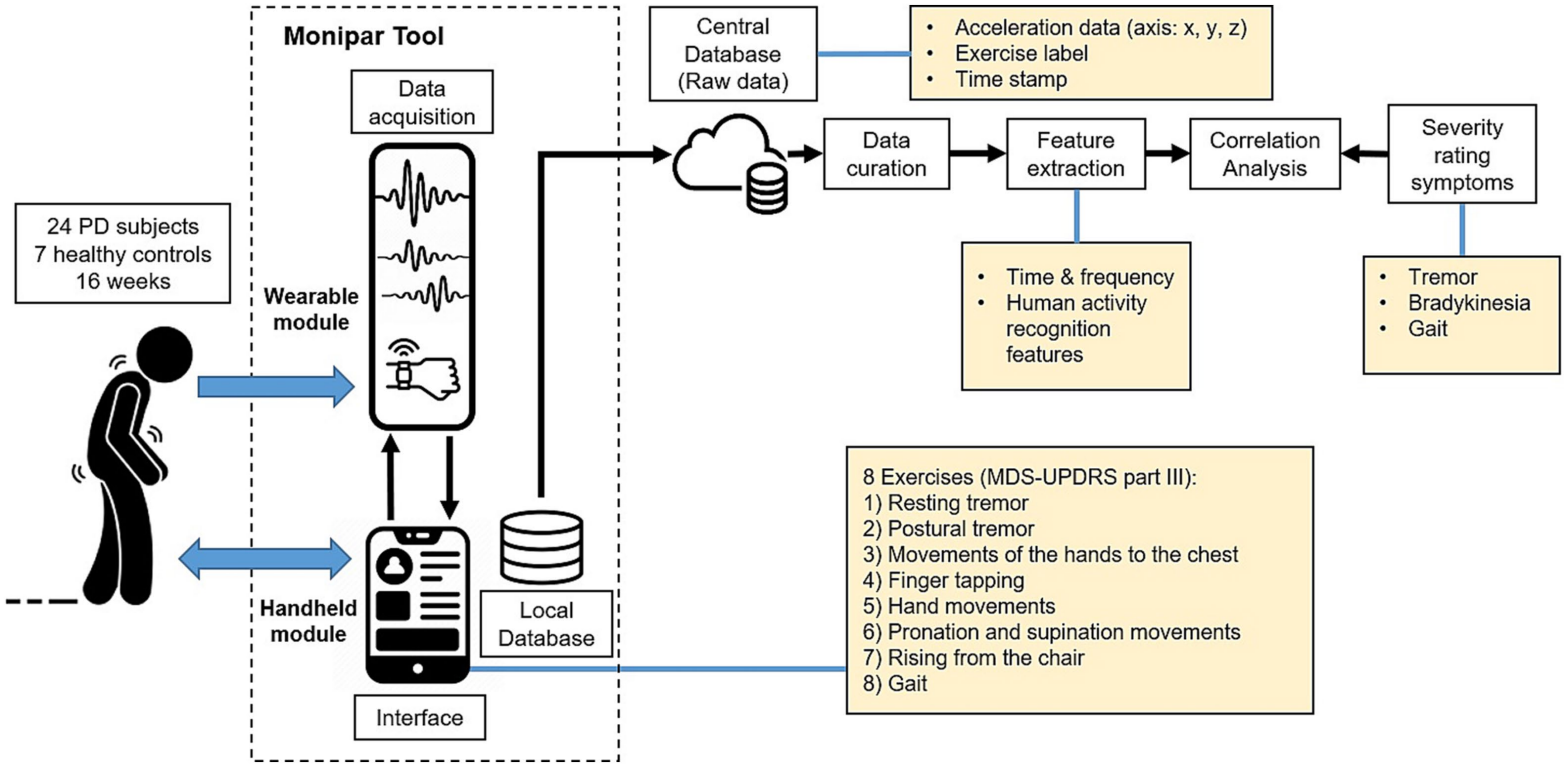
Diagram of the proposed monitoring framework.

The proposed framework is described in Figure 1. This framework employs the Monipar m-health tool to collect data using the built-in accelerometer of an SW during the execution of a set of eight exercises, most of them selected from MDS-UPDRS part III. Data from each SW are stored in a local database and then transferred to a central database for offline analysis. This analysis consists of three stages, namely: data curation, feature extraction, and correlation analysis. The last stage compares the extracted features with the severity rating performed by the MDS-UPDRS scale.

Monipar was developed collaboratively with neurologists and therapists of PD associations and tested in a 4-month study involving 21 early-stage PD patients and 7 healthy control (HC) subjects within the TECAPARK project (33). Participants completed weekly remote motor assessments using Monipar over a 16-week period, starting on different dates. The data collected were then analyzed to assess its potential in monitoring symptoms such as tremors and bradykinesia.

### 2.2 Monipar tool

**Figure 2:**
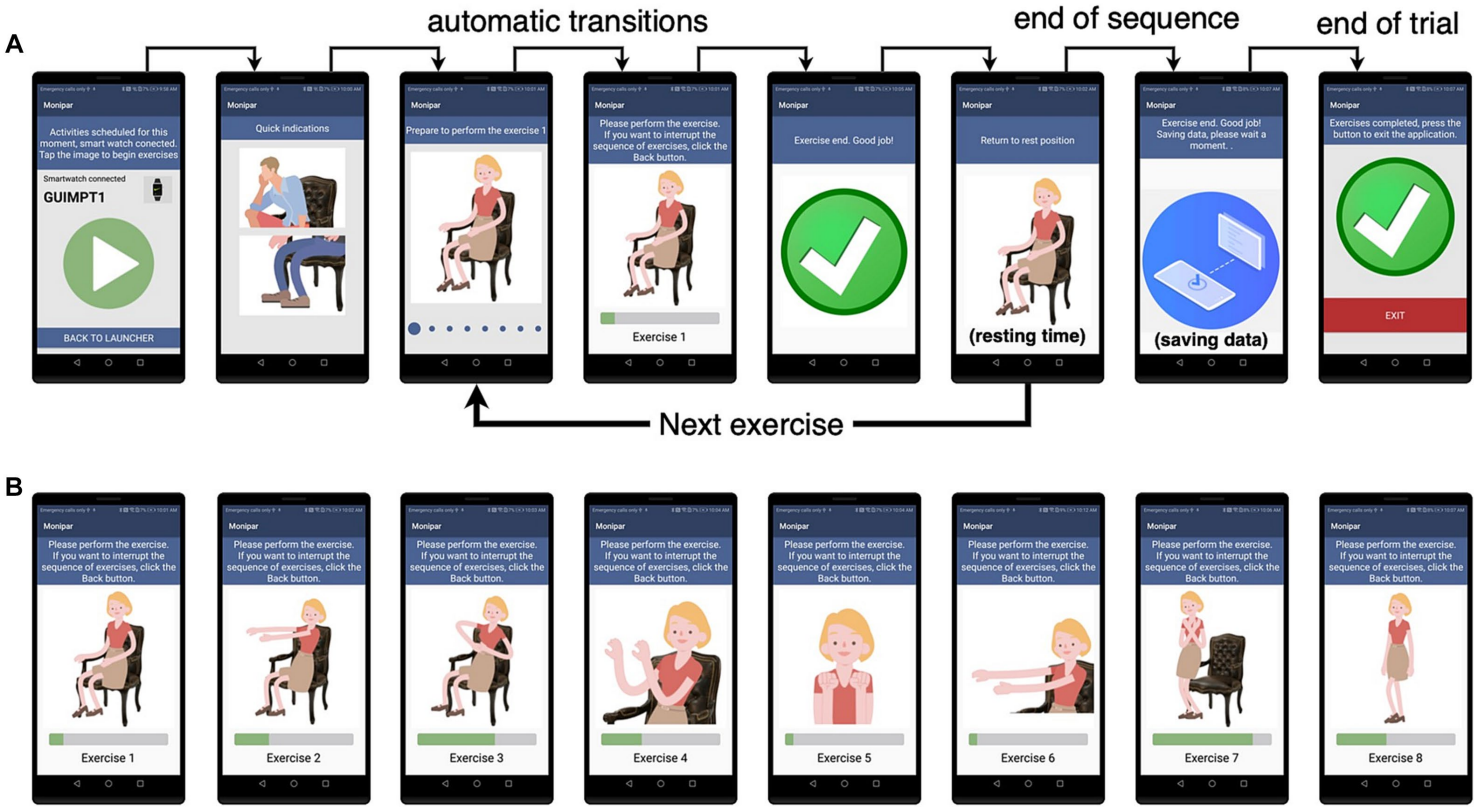
Dynamics **(A)** of the handheld module which guides the user in performing each of the eight exercises proposed **(B)**.

The Monipar tool was developed to facilitate the implementation of the proposed protocol. The application consists of a handheld module (HM) and a wearable module (WM), as shown in Figure 1, which were developed in Android Studio® and installed in the SP and SW, respectively. The HM provides a user interface to guide the patient or caregiver in how and when to perform the proposed exercises using animated images and audio instructions, as shown in Figure 2.

**Figure 3:**
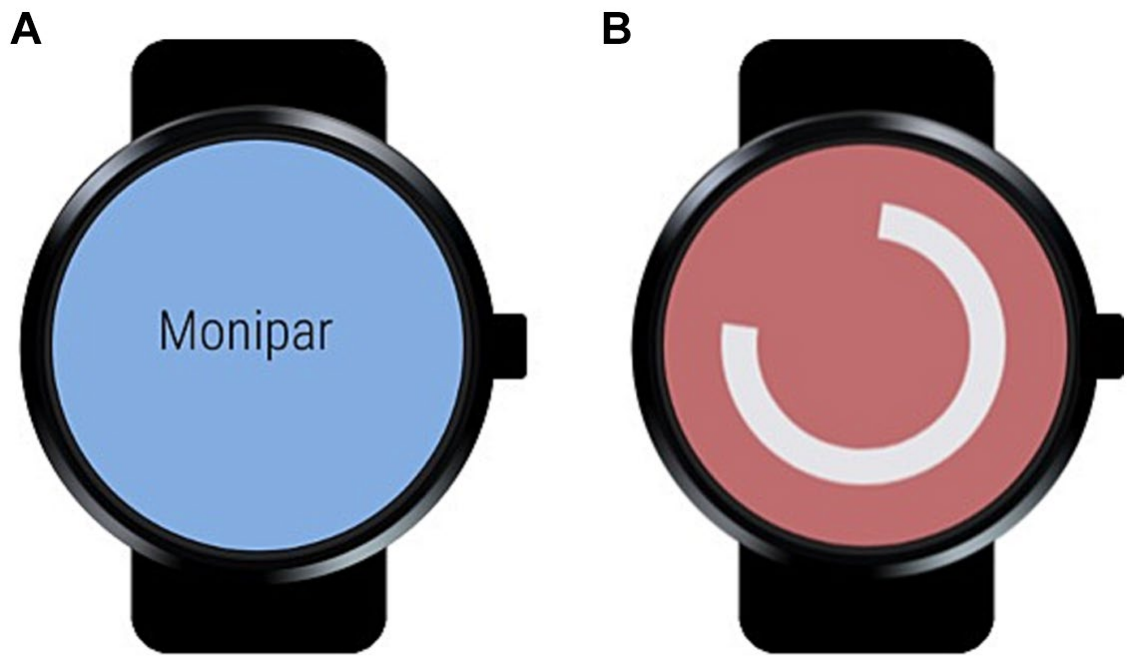
Smartwatch module interfaces: **(A)** Main window; **(B)** Data collection window.

The wearable module (WM) acquires the signals from the built-in accelerometers during the execution of each exercise and then transfers all data to the HM once all exercises have been completed for subsequent analysis. This module was implemented using simple interfaces as shown in Figure 3. Figure 3A indicates that communication with the smartwatch module was established, while Figure 3B indicates that the data recording is activated. Data were first stored in a local database on each smartphone, which was periodically synchronized with a central database using an Internet connection for later processing.

During Monipar execution, the HM sends activation and status indicators to the WM module to automatically label the captured signals with exercise and rest periods tags. In this study, the accelerometer was selected because it is a type of sensor widely used in different smart devices (31). The suitability and accuracy of this sensor for the evaluation of motor symptoms were analyzed in a previous study (34), identifying that the frequency and amplitude configuration allows the collection of voluntary human movement data (<10 Hz) (35), and can analyze the typical frequency range attributed to PD tremors (3.5–7.5 Hz) (36).

For data collection, the accelerometer sampling rate was set at 50 Hz, allowing the analysis of signals with a frequency content of up to 25 Hz according to Nyquist’s Theorem (37). To ensure data consistency, a single-consumer SW model (Tickwatch S2, Mobvoi) and SP (Honor 9 Lite, Huawei) were used in the experimental data collection stage. More in detail, the application was co-designed with 5 therapists from Asociación Parkinson Madrid and a neurologist specialized in PD mainly through discussion meetings and focus groups where we used mock-ups and working prototypes to define the desired functionalities and usability requirements. The interaction with the therapists’ led to improvements in the interface (i.e., the size and shape of the buttons, icons, and legends), the implementation of guidance methods through animated images and voice messages, and the adjustment of the time assigned to the execution of the exercises. The neurologist’s contributions focused mainly on the selection, timing, and sequencing of exercises, the adjustment of resting time intervals, and the setting of the environmental requirements to perform the exercises appropriately. The final working prototype was validated at MIT’s AGELAB with 9 PD subjects to assess its usability and improve its functionality.

### 2.3 Movement exercises

A set of seven exercises was selected from part III of the MDS-UPDRS scale (7), which refers to the “Examination of motor aspects.” An exercise corresponding to repetitive movement performed by stretching the arms and bringing the hands to the chest was included, conforming a set of 8 exercises. Although MDS-UPDRS Part III considers the evaluation of motor competence on each side of the body, in this study, the SW was placed on the patient’s wrist where the greatest presence of motor symptoms was identified according to the clinical indication of the physician who accompanied the participant. For HC subjects, the device was placed on their dominant hand. Also, a rest period was included between each exercise to allow users to relax before the execution of the next task.

The set of exercises was performed weekly and movement data were continuously recorded during each exercise and rest periods. Table 1 shows a summary of the exercises proposed in the experimental protocol and their corresponding section on the MDS-UPDRS scale.

**Table 1:** Selected exercises for the experimental protocol.

Exercise	MDS-UPDRS correspondence	Description
(1) Resting tremor in the upper limbs.	3.17 Rest tremor amplitude.	The patient, while sitting, rests his hands on the arms of the chair and must maintain the posture for 30 s.
(2) Postural tremor of the hands.	3.15 Postural tremor of the hands.	The patient, while sitting, extends his arms in front of him at chest level and holds the posture for 30 s.
(3) Movement of the hands to the chest.	Does not apply (proposed exercise).	The patient, while sitting, stretches his arms and then touches his chest; this exercise is repeated 10 times.
(4) Tapping of the thumb and index fingers.	3.4 Finger-tapping.	The patient, while sitting, should tap the index finger with the thumb 10 times, as fast and wide as possible. The duration of the exercise is 10 s.
(5) Rapid movements of the hands.	3.5 Hand movements.	The patient closes his fist tightly with his arm bent at the elbow so that the palm is shown to the evaluator. The patient should open and close the hand 10 times, as fast and as wide as possible. The maximum duration of the exercise is 10 s.
(6) Pronation and supination movements of the upper limbs.	3.6 Pronation-supination movements of hands.	The patient extends the arm to the front with the palm downward. Then, rotate the palm upward and downward alternately 10 times, as quickly and completely as possible. The maximum duration of the exercise is 5 s.
(7) Arising from a chair.	3.9 Arising from a chair.	The patient, seated in the chair, should cross his arms over his chest and touch his shoulders with his hands; in this position, he proceeds to stand up without separating his arms.
(8) Gait evaluation.	3.10 Gait	The patient must walk at least 7 meters, then turn around and return to the evaluator.

For the execution of the set of exercises, patients were asked to sit in a comfortable chair and performed the set of exercises assisted by a specialist. For one of the experimental groups named supervised group, the execution of the exercises was recorded with a video camera with the permission of the patients for subsequent labeling of the recorded data.

### 2.4 Recruitment

The study was approved by the Ethics Committee of the Universidad Politécnica de Madrid. All participants gave their written consent before participating in the experiment and provided sociodemographic and clinical data related to their condition.

Initially, 25 participants with PD and 8 HC were recruited. However, at the beginning of the data collection, 5 participants (4 PD and 1 HC) withdrew from the study, citing personal reasons. The experimental protocol was carried out with the participation of 21 PD subjects recruited from PD associations in the cities of Burgos, Valladolid, Oviedo (Spain), and Guimarães (Portugal). The inclusion criteria aimed at patients clinically diagnosed with PD in early stages of the diseases (1 to 2.5; average 1.5) according to the Hoehn & Yahr scale (H&Y) (38). Exclusion criteria focused on those with mental illnesses, including dementia, or who had health problems other than PD, which prevented physical activity.

The HC group consists of 7 healthy individuals with similar demographics and gender distribution was recruited in the city of Madrid (Spain). Although the implementation of the experimental protocol for the collection of movement data was similar for all participants, in practical terms, three experimental subgroups were established based on the human and technical capabilities of each association. A detailed overview of the experimental subgroups created for this study is presented below.

(1) Remote group: patients diagnosed with PD who completed the experimental protocol in the PD association they regularly attended. The protocol was carried out under the supervision of a specialist from their PD association who was previously trained by the members of the research team.
(2) Supervised group: patients diagnosed with PD who completed the experimental protocol under the same circumstances as the remote group, but who also allowed video recording of exercise performance for subsequent clinical scoring and data labeling.
(3) Healthy control group: healthy participants who performed exercises supervised by team members of the research project. The protocol for this group was carried out at the facilities of the Universidad Politécnica de Madrid.

Table 2 shows a summary of the demographic characteristics of the study population of the three experimental groups.

**Table 2:** Demographic characteristics of the study population.

	Remote group (*n* = 15)	Supervised group (*n* = 6)	Healthy control group (*n* = 7)
Males/Females	8/7	3/3	3/4
Mean age (SD), years	63.6 (±7.5)	64.2 (±8.2)	64.0 (±5.4)
Hoehn y Yahr (stage = n)	1-stage = 5; 1.5-stage = 1; 2-stage = 8; 2.5-stage = 1	1-stage = 6	–

SD, standard deviation.

### 2.5 Data collection and labeling

The data set collected during the experimental campaign consists of the raw data from the acceleration sensor for each exercise and resting period for each of the 21 patients with PD and 7 HC subjects. During the experimentation, each participant performed the complete set of exercises once a week, preferable on the same day and at a similar time. This will be referred to as a single trial. PD subjects were evaluated in their best ON state, when the medication effectively controls motor symptoms, based on clinical assessments and patient history. Furthermore, throughout the duration of the study, all patients maintained their usual medication regimen.

The data collected was labeled using the annotations generated by the Monipar application for the eight exercises and the resting intervals. To ensure accuracy, a meticulous review of these labels was conducted using MATLAB software (version 2021b), aimed at rectifying any discrepancies or offsets present in the automated labeling of the proposed exercises and the corresponding rest periods.

Furthermore, the clinical scoring of the Supervised group’s data was performed for a trained specialist who meticulously reviewed video recordings from weekly single trial to label the corresponding exercises. For tremor, bradykinesia, and gait scoring, the specialist assessed the severity of six specific tasks following the MDS-UPDRS guidelines. Specifically, the specialist assigned scores ranging from 0 (no impairment) and 4 (severe impairment) to Monipar exercises 1,2, 4, 5, 6, and 8 (see Table 1) corresponding to the MDS-UPDRS tasks 3.17 (Rest tremor amplitude), 3.15 (Postural tremor of the hands), 3.4 (Finger tapping), 3.5 (Hand movements), 3.6 (Pronation and supination movements), and 3.10 (Gait), respectively.

In addition, a continuous labeling strategy was implemented in the resting tremor data. In specific, these data were initially labeled using a method that relied on the analysis of the magnitude within the tremor band (3.5–7.5 Hz). During this analysis, empirical thresholds were set to detect the presence of tremors. Subsequently, the specialist reviewed and corrected these labels by comparing them with the reference video recording for each single trial.

### 2.6 Signal pre-processing and feature extraction

Accelerometer signals collected during the study were pre-processed using a third-order Butterworth high-pass filter with a cutoff frequency of 0.5 Hz to reduce the effect of gravity. From these signals, two sets of features were extracted from the time and frequency domains. Although the time domain features provide high discrimination capabilities without introducing significant increases in computation processing (39), the frequency domain features can describe body movements and represent important characteristics of repetitive movements (40).

The two sets of features extracted from these signals are outlined below.

(1) A set of eight features extracted from the time domain and seven representing the power of specific frequency bands commonly used for the analysis of PD symptoms. These features were extracted from the Euclidean norm (Equation 1) obtained from the triaxial signals of the accelerometer. To compute these features, the entire signal segment that corresponds to each of the proposed exercises was used.


(1) $$a\left(i\right)=\sqrt{a_{x}^{2}\left(i\right)+a_{y}^{2}\left(i\right)+a_{z}^{2}\left(i\right)}$$

Where $a_{x},a_{y},a_{z}$ are the acceleration values corresponding to the x, y, and z axes, respectively.

Table 3 shows a summary of the 15 extracted features, including 8 time- and 7 frequency-domain features to evaluate freezing of gait (41), tremors (42, 43), bradykinesia (43), gait (44), dyskinesia (45) and the band associated with human movements (41). This set of features was extracted only from the supervised group data (6 PD subjects, 46 single trials) that have the MDS-UPDRS evaluation of specific motor task. Furthermore, this feature set was used to perform a correlation analysis with the MDS-UPDRS assessment.

**Table 3:** Features extracted from time and frequency domains.

Domain	Features	Description
Time	Standard deviation	Standard deviation of the raw time series
	Mean	Mean value of the raw time series
	Median	Median of the raw time series
	Percentile 25	25th percentile of the raw time series
	Percentile 75	75th percentile of the raw time series
	Skewness	Skewness of the raw time series
	Maximum value	Maximum value of the raw time series
	Minimum value	Minimum value of the raw time series
Frequency	Freezing of gait band	Freezing of gait band (3–8 Hz) (41)
	Tremor band (4–6)	Tremors band (4–6 Hz) (42)
	Tremor band (3–8)	Extended tremors band (3–8 Hz) (43)
	Bradykinesia and dyskinesia	Bradykinesia and dyskinesia band (0–3 Hz) (43)
	Gait band	Gait detection band (1–3 Hz) (44)
	Dyskinesia (1–4)	Dyskinesia band (1–4 Hz) (45)
	Band power (0–20)	Full band power (0–20 Hz) (41)

(2) A set of 290 features commonly used for automatic human activity recognition (39). These features are frequently used to identify activities of daily living (i.e., sitting, standing, walking), however, in this study, they were used to perform an exploratory visual analysis. This set of features includes time and frequency domain features that were extracted from the raw triaxial signals, the Euclidean norm of the triaxial signals, and the jerk of all previous signals. The reader can refer to (39) for more details on the extracted features. Furthermore, to extract these features, a sliding window of 2.56 s (128 samples) with 50% overlap was used. In specific, this set of features was extracted from the supervised group data (6 PD subjects, 46 single trials with MDS-UPDRS evaluation) and the HC group data (7 subjects, 56 single trials). The resulting feature vectors were labeled with their respective exercise identification and the corresponding MDS-UPDRS score for subsequent selection and analysis. For HC, the data was labeled with the value 0, indicating that there was no impairment in the MDS-UPDRS score. Finally, this set of features was used to perform an exploratory analysis using data visualizations.

### 2.7 Data visualizations

After feature extraction, the set of 290 features was reduced to two features (dimensions) using the t-distributed stochastic neighbour embedding technique (t-SNE) (46). The t-SNE technique is an unsupervised dimensionality reduction tool used to visualize high-dimensional data that have non-linear relationships. In this study, the t-SNE technique was used to perform a visual data analysis to identify any underlying pattern in the data. In specific, two visualizations were generated by applying the brushing technique to highlight the corresponding clinical score performed with the MDS-UPDRS. These visualizations enabled to identify some kind of relationship or trend in the feature set that could be further exploited for the implementation of algorithmic approaches for the detection of specific motor symptoms.

### 2.8 Statistical analysis

A Pearson correlation analysis was performed using the first group of features extracted from the weekly assessment data from the supervised group. The Pearson correlation was chosen in this analysis due to its effectiveness in detecting linear relationships between variables and considers both the magnitude and direction of relationships. These features were compared with the score obtained using the corresponding sections of the MDS-UPDRS. This analysis was performed to identify the features that show the best correlations with the assessment of specific motor symptoms such as tremor and bradykinesia. For this analysis, only data from the supervised group were used, since these data have clinical evaluations carried out through video recordings acquired during the data collection process.

## 3 Results

### 3.1 Data set collected in the experimental stage

Table 4 shows a summary of the number of trials carried out during the experimental stage, as well as how many were collected and lost. The number of individual trials collected for each participant ranges from 2 to 9 weeks (average trials = 6.2). The database collected for this study is publicly available in a Zenodo repository and the data structure can be consulted in (47).

**Table 4:** Summary of the number of trials conducted and data collected.

Group	PD association or location	Performed trials	Collected trials	Lost trials	Percentage of trials lost
Remote	Burgos	20	17	3	15%
	Valladolid	23	16	7	30%
	Asturias	46	39	7	15%
Supervised	Guimarães	46	46	0	0%
Healthy controls	Universidad Politécnica de Madrid	56	56	0	0%
	TOTAL	191	174	17	8.9%

As shown in Table 4 the experimental campaign carried out remotely in the associations of Burgos, Valladolid, and Asturias presented the highest amount of data lost (from 15 to 30%). These data were lost due to storage and communication errors in the prototype of the Monipar application. However, the results of the experiment conducted in Guimarães and the control group show no missing data. This is because the experiment in Guimarães was carried out with an updated version of Monipar, which included redundant data saving to reduce data loss.

### 3.2 Quantitative analysis of the database

The data collected during the experimental stage presents a total of 22 h. These data are divided according to experimental groups, as summarized in Table 5.

**Table 5:** Amount of movement data collected according to the experimental groups.

Experimental group	Number of trials	Hours	Percentage of data
Remote	72	9.1	41.4%
Supervised	46	5.8	26.4%
Control	56	7.1	32.2%
Total	174	22	100%

**Figure 4:**
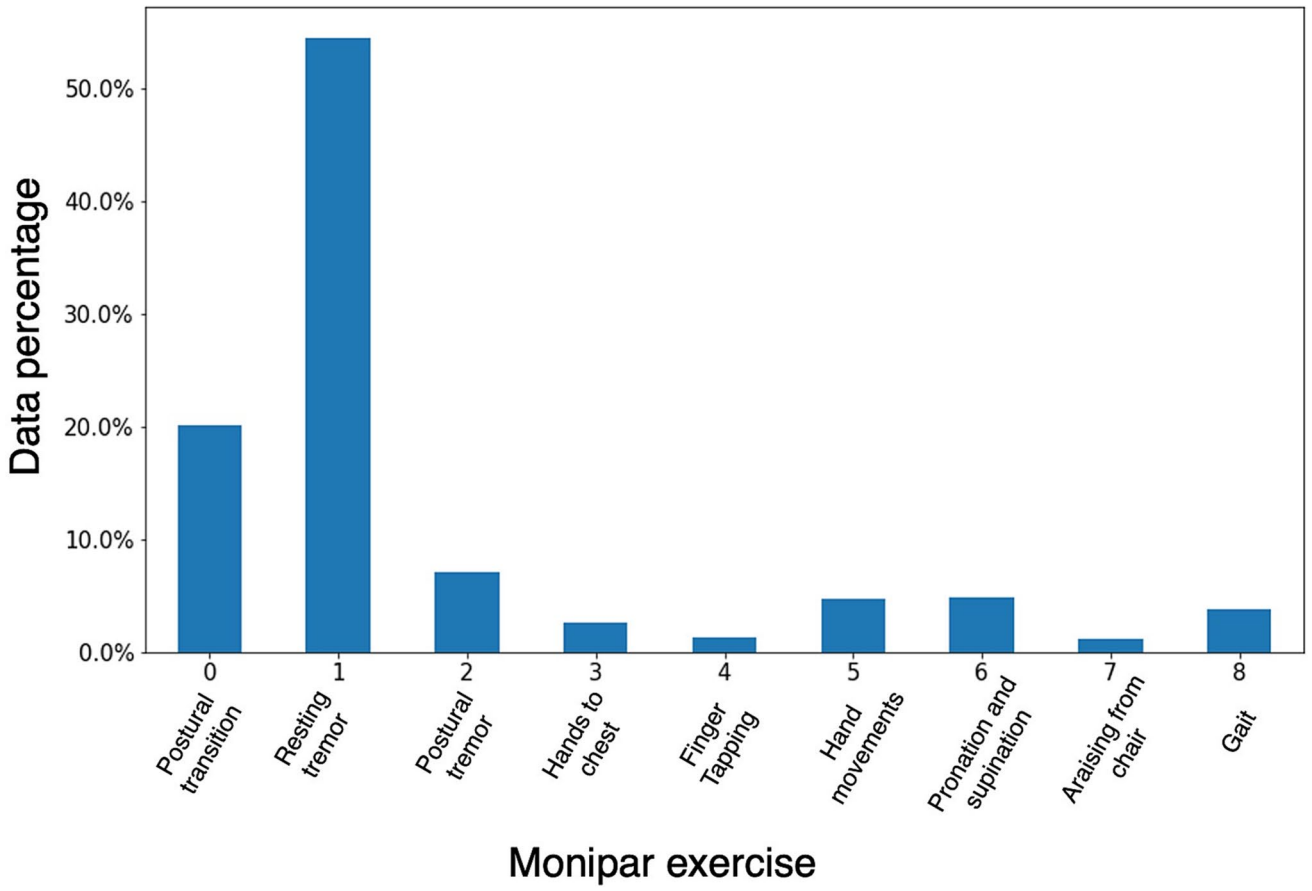
Percentage of data corresponding to the proposed exercises and the postural transition times.

Figure 4 illustrates the distribution of data for various activities and postural transitions. 50% of these data correspond to resting intervals, used to evaluate resting tremors (label 1); while 30% correspond to the execution of the exercises (labels 2–8). Furthermore, 20% of the data were identified as postural transitions (label 0) performed between exercises. The exercise with the least amount of data was the exercise to get up from the chair (1%) (label 7).

### 3.3 Correlation analysis between movement data and the assessment using the MDS-UPDRS scale

**Figure 5:**
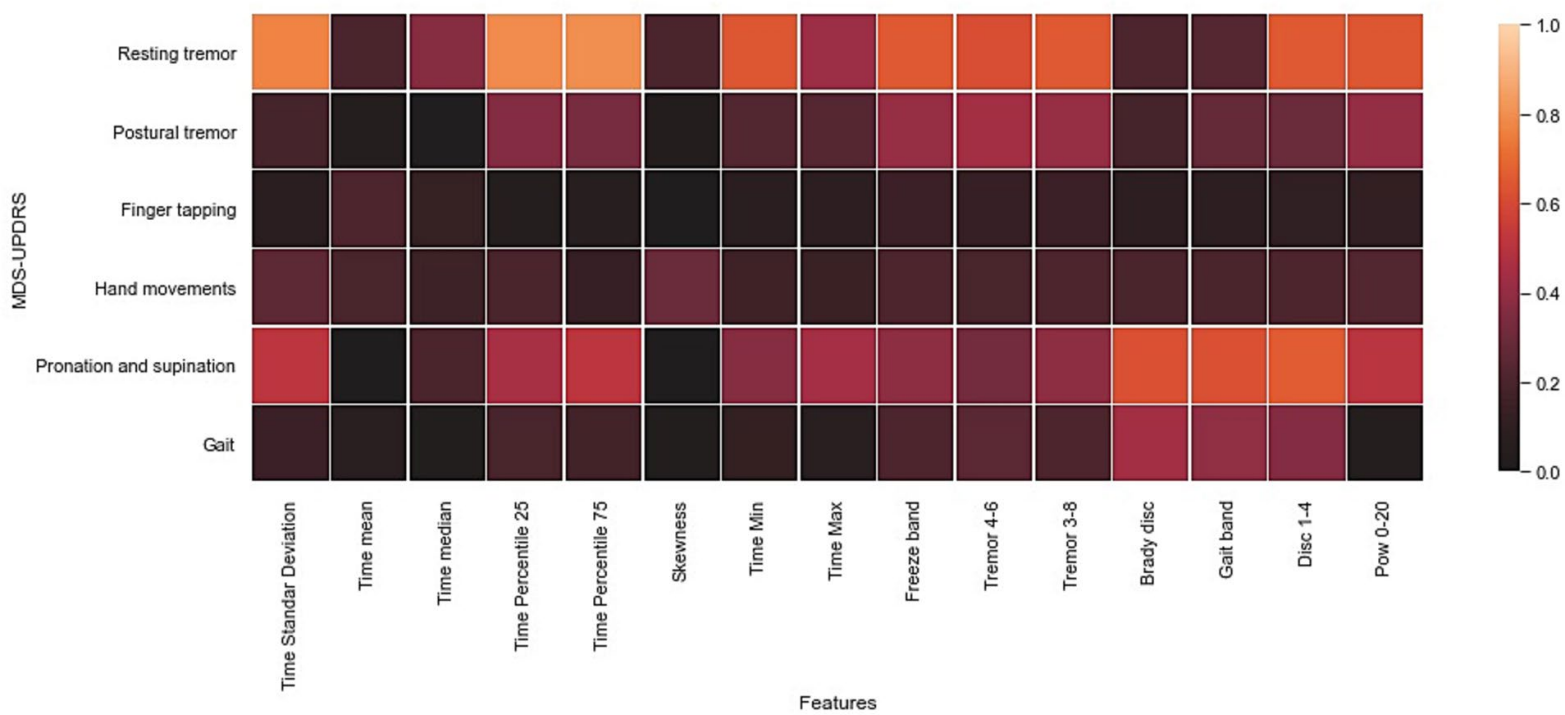
Heat map of the Pearson correlation coefficient of the inter-feature analysis and the UPDRS part III assessment. The values in square grids represent the absolute magnitude of the r value of the correlation analysis.

A Pearson correlation analysis was performed using the set of features indicated in Table 2 and the clinical evaluation that was performed with the corresponding sections of the MDS-UPDRS part III. In this analysis, the data from Exercise 3 (arm movement) were discarded because it does not have a standardized clinical assessment and data from Exercise 7 were also discarded due to the limited amount of data (e.g., 2 s accelerometer signal for each single trial). The selected exercises and their average MDS-UPDRS scores are shown in Table 6. The results of the absolute Pearson correlations are shown in Figure 5 using a correlation matrix.

**Table 6:** Average MDS-UPDRS scores of the selected exercises in the supervised group.

Monipar exercise	MDS-UPDRS item	Average MDS-UPDRS (standard deviation)
1	3.17 Rest tremor amplitude	0.2 (0.49)
2	3.15 Postural tremor of the hands	0.9 (0.68)
4	3.4 Finger tapping	2.4 (0.71)
5	3.5 Hand movements	2.1 (1.04)
6	3.6 Pronation-supination movements of hands	2.5 (0.88)
8	3.10 Gait	1.5 (0.75)

According to Figure 5, the highest absolute correlations were achieved with data from exercise 1 (resting tremor) and with most of the time and frequency features. Furthermore, data from exercise 6 (pronation and supination) showed high correlations with the power of specific frequency bands such as bradykinesia, dyskinesia, and gait bands. Furthermore, the gait and postural tremor data showed moderate correlations with specific frequency bands (e.g., Gait band, freeze band, Bradykinesia and dyskinesia, and tremors bands); these results are expected because gait patterns are commonly used in the evaluation of bradykinesia (48), while the increase in the power of tremors bands have been recognized as clear indicators of the presence of resting, postural, and action tremor (42, 43). The remaining exercises included in the Monipar framework presented a weak correlation with clinical evaluation, probably due to the location of the sensors on the body, which might not be suitable for detecting certain movement patterns, for example, finger tapping.

This correlation analysis shows the potential of data collected using Monipar for the analysis of specific symptoms, including resting tremors and bradykinesia of the upper extremities. For further analysis of data from exercises 1 and 6, Table 7 shows the correlations obtained between the extracted features described in Table 3 and the corresponding clinical scoring (i.e., MDS-UPDRS 3.17 resting tremor; MDS-UPDRS 3.6 pronation and supination movements).

**Table 7:** Results of correlations for the evaluation of bradykinesia and resting tremors.

Feature	Resting tremor (Evaluated using MDS-UPDRS 3.17)	Pronation and supination (Evaluated using MDS-UPDRS 3.6)
	*r* (*p* value)	*r* (*p* value)
Standard deviation (Time)	0.772 (*p* < 0.001)	−0.515 (*p* < 0.001)
Mean (Time)	0.201 (*p* = 0.18)	−0.005 (*p* = 0.97)
Median (Time)	−0.372 (*p* = 0.01)	−0.197 (*p* = 0.19)
Percentile 25 (Time)	−0.792 (*p* < 0.001)	0.461 (*p* = 0.001)
Percentile 75 (Time)	0.804 (*p* < 0.001)	−0.512 (*p* < 0.001)
Skewness (Time)	0.197 (*p* = 0.19)	−0.002 (*p* = 0.99)
Min (Time)	0.644 (*p* < 0.001)	−0.374 (*p* = 0.01)
Max (Time)	−0.424 (*p* = 0.003)	0.450 (*p* = 0.002)
Freeze band	0.653 (*p* < 0.001)	−0.388 (*p* = 0.008)
Tremor band 4–6	0.616 (*p* < 0.001)	−0.315 (*p* = 0.03)
Tremor band 3–8	0.653 (*p* < 0.001)	−0.388 (*p* = 0.008)
Bradykinesia and dyskinesia	0.205 (*p* = 0.17)	−0.622 (*p* < 0.001)
Gait band	0.230 (*p* = 0.12)	−0.620 (*p* < 0.001)
Dyskinesia band 1–4	0.655 (*p* < 0.001)	−0.662 (*p* < 0.001)
Power band 0–20	0.648 (*p* < 0.001)	−0.507 (*p* < 0.001)

The results in Table 7 indicate significant and strong correlations between the clinical score of resting tremor and features extracted from the time domain, including features such as the standard deviation (*r* = 0.772, *p* < 0.001), percentile 25 (*r* = −0.792, *p* < 0.001) and the percentile 75 (*r* = 0.804, *p* < 0.001). Furthermore, significant and moderate correlations (*r* > 0.5) with several frequency bands described in the related literature were also found. Although no clinical explanation can be reported for the high and moderate correlation between the extracted time domain features (standard deviation, percentile 25, and percentile 75) and the clinical evaluation of resting tremors, the authors hypothesize that for this behavior is the result of the absence of movement captured by the sensors during rest periods in the resting tremor data. This absence contrasts with the presence of tremors, leading to signal variations that elevate the values of these time domain features.

For pronation and supination data, significant negative moderate correlations were found between the clinical scoring and time features including standard deviation (*r* = −0.515, *p* < 0.001), percentile 75 (*r* = −0.512, *p* < 0.001), and low-frequency bands such as bradykinesia and dyskinesia (*r* = −0.622, *p* < 0.001), dyskinesia (*r* = −0.655, *p* < 0.001), gait band (*r* = −0.620, *p* < 0.001) and the power band of 0–20 Hz (*r* = −0.507, *p* < 0.001). Furthermore, the moderate correlation between the time domain features (standard deviation and percentile 75) can be attributed to the relationship between the variations in the amplitude and pattern of the movements observed between individuals with motor impairment and those without motor impairment. Although there is no explicit clinical rationale for these time domain features, the findings imply that these features could serve as complementary indicators for implementing multivariate analysis or as a basis for the development of automatic classifiers based on machine learning techniques.

### 3.4 Visualizations generated with the movement data

**Figure 6:**
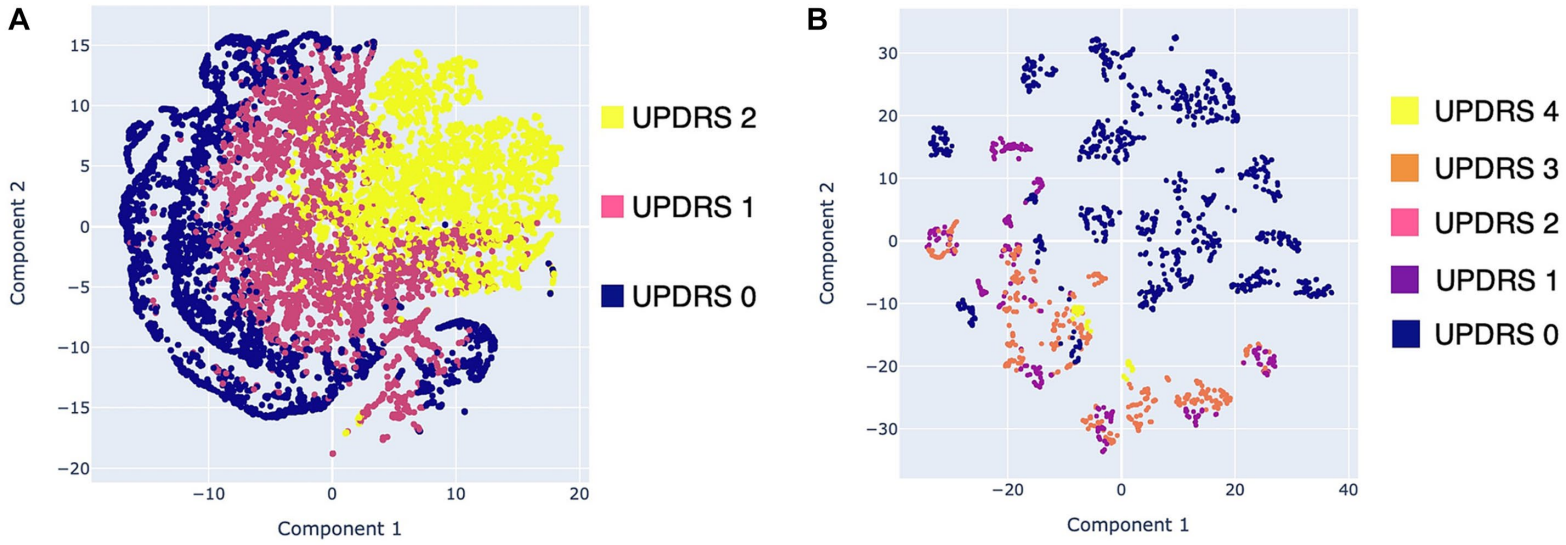
Scatter plots with the data obtained with Monipar: **(A)** Scatter plot of resting tremor data using the MDS-UPDRS 3.17 rating as mapping variable; **(B)** Scatter plot using data of pronation and supination movements using the MDS-UPDRS 3.6 rating as mapping variable.

Visualizations were generated for the resting tremor data (exercise 1) and the pronation and supination movements data (exercise 6). These data were chosen because they presented the highest Pearson correlations with the clinical evaluation in the previous subsection. Figure 6 shows scatter plots of the two components obtained from the data of resting tremor, and pronation and supination movements. In Figure 6A, the tremor rating performed according to Section 3.17 of the MDS-UPDRS scale was used as the mapping variable. In Figure 6A only data from the Supervised group were used corresponding to 46 single trials from 6 subjects with PD. Moreover, in Figure 6B, the bradykinesia rating performed according to Section 3.6 of the MDS-UPDRS scale was used as the mapping variable. In this visualization data from the Supervised and HC groups were used that correspond to 102 single trials from 13 subjects (6 PD and 7 HC).

Figure 6A shows three clusters corresponding to the MDS-UPDRS scoring with a slight degree of overlap. These overlaps are expected, as the severity of symptoms is continuous, rather than the discrete scoring system proposed on the MDS-UPDRS scale (49). Figure 6B shows two clusters of data belonging to healthy control patients (bradykinesia: 0) and patients with PD (bradykinesia: 1–4). However, a high overlap between MDS-UPDRS scores is identified.

Overall results of the visualizations generated using data from resting tremor and supination movements suggest the feasibility of implementing automatic classifiers. Moreover, the visualization shown in Figure 6B suggests that it is viable to implement automatic classifiers for discrimination between healthy subjects and PD patients. However, more data should be needed to determine whether it is feasible to detect different degrees of bradykinesia using the proposed framework.

## 4 Discussion

### 4.1 Main results

The results indicate that the proposed framework based on the execution of standardized exercises monitored using off-the-shelf devices can provide useful data to derive digital indicators to monitor motor symptoms in the upper extremities. In particular, data collected from exercises used to assess tremors and bradykinesia allowed the extraction of indicators that show high correlations with clinical evaluation. Although indicators extracted from exercises such as gait, finger tapping, and hand movements (i.e., open and close hands), presented weak and moderate correlations, which may be attributed to the location of the sensors on the wrist that difficult the acquisition of specific movement patterns, for example, those mainly produced for the fingers or those produced when opening and closing the palms of the hands.

Along with these results, the data collected during the experimental stage presented different percentages of data loss depending on the experimental group. In specific, the remote group presents the higher data loss rate, ranging from 15 to 30%, while the supervised group and the HC (both performed in a completely supervised setting) do not show any data lost. Despite this behavior, the null lost rate in the Supervised and HC group was also influenced by the implementation of security actions, such as redundant data saving in both devices (i.e., SP and SW). Furthermore, guidance in the performance of standardized exercises, through images and voice prompts, seems to be a feasible method to ease the implementation of movement data collection protocols performed remotely.

The overall results suggest that consumer SW in conjunction with SPs can be used as an economic and ergonomic solution to acquire useful data to monitor bradykinesia and resting tremors using two specific tasks proposed in the MDS-UPDRS scale. Despite these results, the remaining proposed exercises exhibit moderate (e.g., postural tremor and gait) and weak (e.g., finger tapping and hand movements) correlations with the clinical assessment. This situation highlights the need to develop novel data collection methodologies and data processing strategies to enable remote monitoring of relevant symptoms such as gait, stiffness, and postural stability. Specifically, to improve gait and sit-to-stand assessment, the results suggest the importance of incorporating complementary sensors strategically positioned on the body parts such as the waist or legs. In addition, specific activities, such as finger tapping, may require the use of specific sensors to provide a more accurate representation of specific movements that are considered in the clinical evaluation.

### 4.2 Comparison with previous work

This study provides evidence of the feasibility of off-the-shelf SW and SP to provide a cost-effective, convenient, and unobtrusive solution for data collection aimed at monitoring cardinal motor symptoms such as tremors and bradykinesia. These findings complement the results reported in the related literature in which the use of commodity SW (24, 25), research-grade wrist devices (21, 22), and SP as part of multimodal systems (26, 29) have reported feasible solutions for collecting data to monitor motor aspects in laboratory, in-clinic and unsupervised settings.

Furthermore, the correlation analysis based on data collected by Monipar and the clinical assessment reveals moderate to strong correlation; in specific, higher correlations were identified using data from resting times and pronation and supination movements. These findings suggest that an accurate selection of specific and representative tasks can be the basis for the development of abbreviated and robust motor monitoring protocols aimed at improving patient adherence, such as the one proposed in (22, 24), where wrist rotation movements and arms resting captured with SW were used to detect short-term motor fluctuations and long-term responses to therapies. In specific, the results reported in (22) using a commercial SW (Verily Study Watch) present a similar correlation with the MDS-UPDRS Part III ratings to those obtained in this study (i.e., Spearman rank correlation for rest tremor ρ = 0.70; bradykinesia ρ = −0.62).

Additionally, this study shows the potential of off-the-shelf SW for the acquisition of movement data in patients in the early stages of PD (H&Y ≤ 2.5), where the presence of motor manifestations is generally mild and, therefore, an accurate monitoring of digital variables such as the frequency and amplitude of tremors can require a high sensor sensitivity (50, 51).

Finally, the visualizations generated using the data collected by Monipar show the potential of these data for the development of different algorithms that can be used to monitor tremors or bradykinesia. Examples of these applications were described in previous studies using the same database (34, 52).

### 4.3 Limitations

This study has some limitations that provide directions for future research. These limitations include the small sample size of healthy controls compared to participants with PD (21 PD and 7 HC). Also, the fact that this study considers only PD subjects in the early stages of the disease, therefore conducting a larger-scale longitudinal data collection may provide a better representation of the broad spectrum of motor symptoms and manifestations. Moreover, conducting larger-scale experiments can allow the evaluation of the cost-effectiveness and scalability of these technologies to support their adoption in clinical management (14).

Additionally, other relevant cardinal motor symptoms such as rigidity and postural instability were not assessed in this study due to the inherent difficulty in monitoring this symptom using a single accelerometer. However, the inclusion of specific task and complementary sensors can support the development of monitoring solutions to assess multiple motor manifestations.

Finally, this study focused only on evaluating the ability of the Monipar tool to acquire data intended for the assessment of motor competence. Including other modules to assess non-motor symptoms can contribute to providing a broader overview of the health state of a PD subject.

## 5 Conclusion

The implementation of the proposed framework to monitor motor symptoms has generated a database that presents a high capability for the detection of specific motor symptoms such as resting tremors and bradykinesia. This framework was implemented through the development of an *ad-hoc* tool named Monipar that uses commodity SW for the acquisition of motion signals during the execution of standardized exercises.

During the data collection stage, the use of Monipar simplified data collection tasks and the implementation of experimental protocols such as the one proposed in this study, which was based on the performance of selected MDS-UPDRS exercises. The use of guides to perform the set of exercises supported by a graphical interface with animated images and voice instructions has shown a feasible method to facilitate the understanding of the assigned motor tasks and improve usability.

The correlation analysis performed using the data collected by Monipar shows moderate to strong correlations between several indicators and two specific MDS-UPDRS exercises designed to evaluate resting tremors and bradykinesia in the upper extremities. These correlations revealed the most representative features for the analysis of specific symptoms such as tremor and bradykinesia. Additionally, visualizations created using the t-SNE method and tremor and bradykinesia show the generation of clusters with a small (yet expected) amount of overlap between the MDS-UPDRS scores.

Overall results of this study suggest that Monipar can be used as a complementary tool for data collection and follow-up of specific motor disorders in PD, at least in early-stage patients, providing a feasible and cost-effective solution for remote and continuous monitoring of the evolution of cardinal motor symptoms. In future applications, the information generated by this kind of monitoring system can be used to improve disease management, support decision-making, and become part of integrated telemedicine and digital health systems. Future work should address the development of novel algorithms and feature extraction strategies to develop robust methods to monitor specific motor manifestations. Furthermore, standardization of data collection methodologies is important to facilitate the comparability and integration of digital outcomes to provide a comprehensive overview of the disease to allow better clinical care, assessment, and monitoring of PD according to the roadmaps proposed in (14, 30).

## Data availability statement

The raw data supporting the conclusions of this article will be made available by the authors, without undue reservation. The datasets generated for this study can be found in the Zenodo repository, https://doi.org/10.5281/zenodo.8104853.

## Ethics statement

The studies involving humans were approved by Institutional Review Board of the Universidad Politécnica de Madrid (date of approval: 18 June 2018) and the Ethics Committee of the University of Minho with the document identification CE.CSH 031/2018 (date of approval: 11 December 2018). The studies were conducted in accordance with the local legislation and institutional requirements. The participants provided their written informed consent to participate in this study.

## Author contributions

LS: Conceptualization, Data curation, Investigation, Methodology, Software, Visualization, Writing – original draft, Writing – review & editing. CP-F: Conceptualization, Formal analysis, Investigation, Writing – original draft. NC: Conceptualization, Funding acquisition, Project administration, Supervision, Validation, Writing – review & editing. SC: Conceptualization, Formal analysis, Validation, Writing – original draft. PA: Formal analysis, Project administration, Resources, Supervision, Writing – review & editing. MG: Formal analysis, Methodology, Validation, Writing – review & editing. CL: Formal analysis, Investigation, Writing – review & editing. JL: Formal analysis, Resources, Supervision, Writing – review & editing. GA: Conceptualization, Formal analysis, Funding acquisition, Project administration, Writing – review & editing. IP: Conceptualization, Formal analysis, Project administration, Validation, Writing – review & editing.
